# Postharvest Handling and Storage Strategies for Preserving Jujube (*Ziziphus jujuba* Mill.) Fruit Quality: A Review

**DOI:** 10.3390/foods14193370

**Published:** 2025-09-29

**Authors:** Muqaddas, Li Mengaya, Mian Muhammad Ahmed, Syeda Maira Hamid, Xiang Yanju, Muhammad Asim, Pu Yunfeng

**Affiliations:** 1 College of Food Science and Engineering, Tarim University, Alar 843300, China; muqaddas031@gmail.com (M.); 15637081398@163.com (L.M.); xiangyanju@163.com (X.Y.); 2 College of Life Science and Technology, Tarim University, Alar 843300, China; mian.ahmed1@outlook.com (M.M.A.); smairahamid@gmail.com (S.M.H.); 3 Production & Construction Group Key Laboratory of Special Agricultural Products Further Processing in Southern Xinjiang, Alar 843300, China; 4 National Key Laboratory for Germplasm Innovation and Utilization of Horticultural Crops, College of Horticulture and Forestry Sciences, Huazhong Agricultural University, Wuhan 430070, China; chasim691@gmail.com

**Keywords:** Jujube (*Ziziphus jujuba*), shelf-life extension, non-thermal preservation, edible coatings, cold plasma technology, microbial control

## Abstract

Jujube (*Ziziphus jujuba* Mill.) is a nutritionally rich and economically significant fruit, highly valuable for its flavor, bioactive compounds, and therapeutic properties. However, it is highly perishable and has a short postharvest lifespan. This review aims to provide knowledge for preserving quality and improving postharvest storage by integrative strategies aimed at extending the shelf life of jujube. The literature was collected from recent peer-reviewed studies on postharvest physiology and handling technologies of jujube fruit. Key physiological factors, influencing postharvest deterioration such as water loss, softening, browning, and decay, are discussed, along with the underlying biochemical and enzymatic mechanisms driving quality decline. Conventional strategies such as cold storage, MAP, and CA effectively slow respiration, delay reddening, and extend storage up to 2–4 months, while emerging approaches such as ozone and cold plasma treatments reduce microbial decay and maintain antioxidant activity. Edible coatings like chitosan, aloe vera, and composites cut weight loss by 20–40%, and chemical regulators such as 1-MCP and calcium dips further delay ripening, preserve firmness, and enhance postharvest quality. Emphasis is placed on integrating innovative technologies with physiological insights to optimize storage conditions, control microbial contamination, and maintain nutritional integrity. The significance of this review lies in integrating physiological insights with innovative preservation methods, offering practical guidance for researchers, growers, and industry stakeholders to achieve sustainable, safe, and market-oriented solutions for jujube storage.

## 1. Introduction

Jujube (*Ziziphus jujuba* Mill.), also known as Chinese date, is a major domesticated fruit tree species with ecological, social, and substantial economic significance. In China, where the cultivation area exceeds 3 million hectares with an annual yield of over 7 million tons, accounting for nearly 98% of global production, it has been cultivated for more than 4000 years [1]. Other countries where jujube is cultivated include those in North Africa, Iran, South Korea, United States, Israel, and several Middle Eastern countries [2]. Jujube is a highly nutritious fruit renowned for its rich composition of amino acids, ascorbic acid, triterpenic acids, polysaccharides, flavonoids, phenolic acids, alkaloids, saponins, and essential minerals [3,4]. Fresh jujube also provides carbohydrates, vitamins, organic acids, and dietary fiber, forming a comprehensive nutritional system that synergistically supports human health [5]. Owing to this diverse composition, jujube exhibits remarkable biological activities, including antioxidant, anti-inflammatory, antibacterial, antitumor, anti-diabetic, antihypertensive, and anti-cancer properties [6,7]. Scientific evidence has further demonstrated its therapeutic potential, with extracts shown to reduce inflammation and combat obesity [8], regulate blood glucose levels [9], and even induce apoptosis in T-cells in leukemia [10]. It is also recognized as a low-calorie fruit with additional health benefits, such as stimulating appetite, improving digestion and gut health, lowering blood pressure, and boosting immunity [6]. Beyond its medicinal and nutraceutical value, jujube fruits are prized for their sensory qualities, including thin skin, thick flesh, crisp texture, juiciness, and a well-balanced sweet–sour flavor, which contribute to strong consumer appeal [5]. Typically, available fresh from January to April, they can also be sun-dried or powdered to extend availability for off-season use [6]. Due to its abundant bioactive metabolites and unique characteristics, jujube holds great promise for the development of functional foods, pharmaceuticals, healthcare products, and cosmetics [4,7].

The economic importance of jujube is substantial and growing, driven by rising consumer awareness of its health benefits. In China, the epicenter of its production and consumption, jujube is not merely a fruit but a cornerstone of traditional medicine and a valued cultural symbol, integrated into various culinary applications from teas and soups to snacks and wines. This deep-rooted domestic demand ensures a stable and high-value market. Internationally, jujube is gaining traction as a “superfruit” in health-conscious markets in North America and Europe, where it is sold fresh, dried, or as a functional ingredient in health foods, supplements, and nutraceuticals. This expanding global consumer demand translates directly into economic incentive for producers; premium-quality fresh jujube can command significantly higher prices than processed forms, but this is contingent upon overcoming its perishable nature. Therefore, the economic losses due to postharvest spoilage are not just a technical issue but a critical market barrier. Effective preservation strategies directly enhance profitability by enabling access to distant, high-value markets and reducing waste, thereby strengthening the economic resilience of the jujube industry globally.

Fresh jujube fruit is favorite amongst food candidates for its crisp texture, sweet taste, and unique nutritional value. However, it is also highly perishable and lasts less than one week at ambient temperature when fully matured [4]. Postharvest losses are large due to the impact of physiological ripening, senescence, chilling injuries, mechanical damage, and microbial decay occurring individually or in combination. These factors contribute to cumulative quality deterioration, i.e., including water loss, softening, browning, alcoholic fermentation, surface pitting, and decomposition [5]. Water evaporation leads to weight loss and reduced textural quality [11], while degradation of pectin and cell wall components gradually decreases firmness [12]. Browning caused by membrane lipid peroxidation, together with fungal and bacterial infections, further accelerates quality decline [13,14,15]. To address these issues, several postharvest technologies have been employed, including cold storage and controlled/modified atmosphere systems to reduce respiration and water loss, edible coatings and calcium treatments to maintain firmness, and innovative approaches such as ozone, UV-C irradiation, and CAP to control microbial contamination and delay browning. Collectively, these strategies aim to preserve nutritional and sensory quality while reducing economic losses, underscoring the need for integrated handling and storage solutions to extend the shelf life and marketability of jujube fruit.

Although several studies have discussed the postharvest preservation of jujube, most have focused on individual methods such as cold storage, edible coatings, or controlled atmospheres in isolation. There is a lack of comprehensive analysis that integrates physiological mechanisms of deterioration with both conventional and advanced preservation strategies. Moreover, little attention has been given to linking technological interventions with the underlying physiological and biochemical processes that govern postharvest deterioration. This review aims to fill that gap by providing a holistic overview of postharvest management approaches for jujube, ranging from harvesting, sorting, cold storage, packaging, sanitation, and chemical treatments to emerging non-thermal and eco-friendly innovations. By comparing these traditional and emerging methods, this review aims to clarify their effectiveness, limitations, and potential for integration into commercial supply chains. The scope is thus focused on identifying best practices, highlighting recent advances, and outlining future research directions to enhance the shelf life, quality, and marketability of jujube fruit. By linking physiological mechanisms of quality deterioration with targeted preservation techniques, this review provides new insights into how synergistic applications can optimize storage outcomes. It further identifies research gaps and future directions, serving as a resource for researchers, growers, and industry stakeholders seeking sustainable, safe, and commercially viable postharvest management of jujube fruit.

## 2. Postharvest Physiology and Deterioration Mechanism

**Figure 1:**
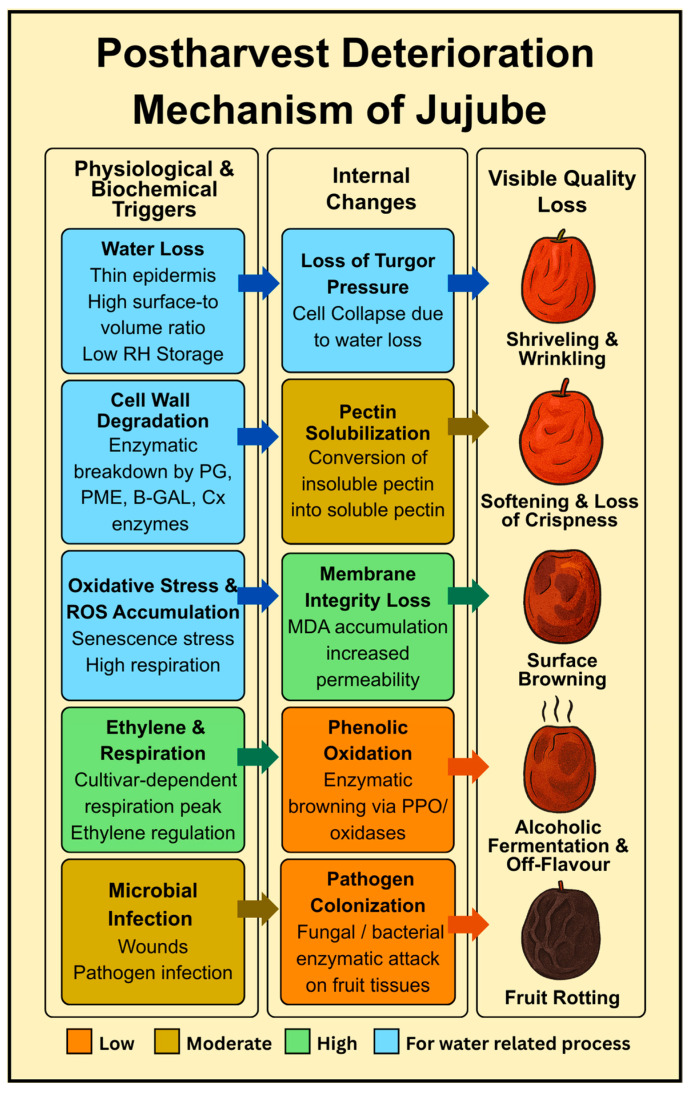
Postharvest physiology and deterioration mechanisms in jujube.

Postharvest physiology of jujube fruit is controlled by a complex interaction of biochemical, physical, and pathological factors (Figure 1). These processes begin immediately after the harvest and extend to postharvest storage, handling, and marketing of the produce. A comprehensive understanding of these physiological processes and pathways of deterioration is necessary to develop effective postharvest treatment to increase shelf life, maintain fruit quality, and reduce postharvest losses. Fruit maturity stage at the time of harvesting is the basic aspect of proper storage and quality parameter of the postharvest jujube fruit [16]. Physiological maturity refers to the point at which the fruit has completed its development on the tree and is capable of ripening off the plant, while commercial maturity is often determined by sweetness, visual cues, and firmness preferred by consumers.

Fruits become softer after reaching the physiological maturity stage and beginning of senescence as pectin is broken down and structural alterations in the polymeric networks of the cell walls occur, causing cell compartmentalization and the loss of rigidity. Consequently, fruit firmness decreases gradually [17]. The timing of harvest is therefore essential, because it directly affects shelf life, maturity profile, and susceptibility to postharvest disorders. The maturity of jujube fruit in winter is mainly determined on the basis of its peel color [18]. The proactive color transitions from green to yellow, half-red, and ultimately red, aligning with the green fruit stage, white maturity (WM), half-red maturity (HRM), and red maturity (RM), respectively [18]. To enhance shelf life, one of the common harvesting stages of winter jujube is the WM stage, followed by storage under low-temperature conditions for some time and then marketing [19].

On the other hand, delayed harvesting of fruit can induce early softening and increase susceptibility to decay, resulting in enormous postharvest loss and short storage life [20]. Another key indicator that affects shelf life and storage potential of fruits is the rate of respiration. The extent of respiration is another major factor that determines postharvest performance of winter jujube [21,22]. Nevertheless, there is ongoing debate over whether winter jujube falls into the climacteric or non-climacteric category [23]. Certain varieties show climacteric traits [24,25], characterized by a moderate peak in respiration and ethylene production during ripening, while others exhibit non-climacteric behavior with minimal change in respiration rate [16,26,27,28]. For instance, the respiration rate of ‘Dongzao’ cv. of jujube reduces initially, followed by a moderate increase toward the end of storage [23]. Similarly, the respiration rate increases significantly during storage of ‘Hupingzao’ and ‘Huizao’ cvs., corresponding with the senescence of fruits and the loss of quality [29]. It is usually characterized by low and stable ethylene production throughout the ripening of the fruit until the full maturation stage is reached, when its production rises significantly [23]. Furthermore, treatment with 1.0 μL/L 1-methylcyclopropene (1-MCP) reduces ethylene production and respiration rate in WM fruits [23]. The results indicate that the jujube fruits are mainly characterized as a non-climacteric type of ripening in which the role of ethylene seemed insignificant in the regulation process of ripening but also was critical in retaining normal ripening processes. Furthermore, treatment with 1.0 μL/L 1-methylcyclopropene (1-MCP) suppressed ethylene production and respiration rate in WM fruits [23]. The results indicate that the jujube fruits mainly performed non-climacteric ripening in which the role of ethylene seemed insignificant in the regulation process of ripening but also was critical in retaining normal ripening processes.

One of the most noticeable and obvious signs of postharvest losses in jujube is water loss, which severely affects both the appearance and texture of the fruit. Excessive moisture loss results in shrinkage and weight reduction, diminishes firmness, and significantly reduces the marketability of jujube fruit [5]. Jujube fruit is particularly susceptible to transpiration due to its comparably thin epidermis and large surface-to-volume ratio. Moisture loss is also catalyzed by external factors such as high storage temperature, low relative humidity, and poor ventilation conditions [29]. Skin permeability also influences the fruit’s ability to retain water. As the water evaporates, the intracellular pressure drops, causing the cell to collapse and the fruit to lose its turgidity, and become leathery and wrinkled, which are two of the most significant defects to affect the consumer-accepted indicators of quality in a fruit [30]. Another frequent postharvest textural change is softening, where there is a gradual loss of firmness. The trend of decrease in the firmness of jujube fruit at room temperature is always evident as long as storage proceeds [16]. Specifically, the decrease in firmness is related to the breakdown of water-insoluble pectin to water-soluble forms [31].

Biochemical and enzymatic alterations in storage are also important in defining the shelf life and nutritional quality of jujube fruit. Ripening is associated with changes in the secondary metabolism, which includes phenolic compounds, flavonoids, and antioxidants. These phytochemicals tend to decrease with prolonged storage, mostly through oxidative processes and enzyme-based reactions [32]. Senescence also results in membrane structural and functional impairment, mainly due to over-formation of ROS and MDA [33]. MDA can be used as a reliable indicator of oxidative damage in plant tissues; it is a major end product of membrane lipid peroxidation [34]. Another significant postharvest issue is the browning of the pericarp, primarily caused by enzymatic oxidation. The oxidase activity shifts these phenolic substrates into colored quinones [35]. As ref. [36] illustrated, membrane lipid peroxidation disrupts cellular compartmentalization, permitting phenolic materials to react with oxidative enzymes to form browning enzymes. ROS accumulation exacerbates this process, promoting further lipid peroxidation and destroying membrane fluidity, integrity, and functionality [37].

Moreover, postharvest softening is a critical process of cell wall degradation in winter jujube. Pectin, which imparts intercellular adhesion and mechanical strength, is projected into soluble forms by pectin-degradation enzymes such as PG, α-L-Af, pectin methyl esterase (PM), and β-Gal. Similarly, cellulose, the most important structural backbone of the cell wall, is hydrolyzed to monosaccharides by Cx and β-Glu [38,39,40]. Such enzyme-related reactions collectively lead to loss of firmness, softening of tissues, and an overall decline in fruit quality.

## 3. Postharvest Handling Practices

### 3.1. Harvesting Techniques

One of the major factors that determines the quality of jujube fruits and enhances shelf life is optimum time of harvesting the fruit. Fruits must be harvested at the physiological maturity stage, which is indicated by change in color, e.g., green to yellow/red, depending on the variety [41]. When harvesting occurs at the right time, it ensures a high amount of nutritional value and healthy resistance to the postharvest diseases [42]. To limit postharvest losses, jujube should be harvested in the cooler hours of the day, since high field temperatures promote faster respiration and accelerate fruit senescence [16]. To obtain the best flavor and quality, fruits should always be harvested in the morning hours when temperatures are low.

Winter jujubes have been hand-picked traditionally by knocking the trees, followed by sorting and packing the fallen fruits [43,44]. The technique is labor-intensive and resource-intensive, and can cause mechanical damage to the fruits. Manual harvesting, which is mostly used in fresh consumption, is the most desirable cultural approach since it causes minimal bruising and skin damage. Although labor-intensive and time-consuming, it provides another opportunity, that is, selective and careful harvesting of ripe fruits and handling, thus preserving quality.

Mechanical harvesting methods like vibration harvesting have been developed to enhance the efficiency of fruit harvesting. In such an approach, the tree is shaken mechanically to make the fruits fall and they are collected using catching materials [45]. This method reduces manpower and reduces harvesting time, although it might increase the damage to fruits compared to using manual methods. To overcome these challenges, ref. [46] built a specialized harvesting and sorting device for winter jujubes. The device integrates a vibration system to shake off the fruit, an umbrella-shaped catch system to avoid bruising when the fruit falls, and a sorting sequence to help handling after harvesting. The efficiency of such systems depends on tree characteristics; for example, the displacement is dependent on the elevated position of the vibrating point. Smaller diameters, the diameter of the trunk, and the elevated positions in general lead to maximum displacement. To make the process of mechanical harvesting successful, it is important to maintain an optimum vibration frequency and amplitude to give high sorting efficiency and reduce the potential injury caused to the fruits.

### 3.2. Sorting and Grading

The commercial value of jujube fruit and consumer acceptance totally depend on two critical postharvest operations, sorting and grading, which directly determine the market quality. Fruit size, color, and the presence or absence of surface blemishes are the standard grading criteria of jujube. Size grading provides uniformity, which is particularly important for packaging efficiency and for fulfilling specific market demands, as larger fruits often provide higher prices in premium markets. Color is another important indicator, serving as a visual cue for ripeness and internal quality. In jujube, cultivars with uniform bright red or yellowish-green shades depending on the maturity stage are most desirable for fresh consumption. The evaluation of blemishes such as mechanical injuries, insect damage, or disease spots is equally important, since such defects not only reduce visual quality but also accelerate spoilage by increasing susceptibility to microbial invasion. Grading fruit quality enhancement before sale notably increases its market value [47].

Effective sorting and grading play a key role in enhancing both consumer satisfaction and commercial outcomes. Uniform, coherent, and defect-free fruit lots strengthen brand reputation, enhance consumer satisfaction, and encourage repeat purchases. On the other hand, inconsistent or substandard grading may lead to shipments of uneven quality, undermining buyer confidence and resulting in economic losses. Proper grading also helps efficient downstream operations such as packaging and storage. Top-quality fruits are prioritized for long-term storage, while lower-grade produce is utilized for immediate processing or local consumption. Mechanical and optical grading systems incorporating machine vision and spectral analysis have been developed to solve limitations in manual grading which is labor-intensive and subject to human error. These advanced systems increase grading precision, reduce labor costs, and certify consistency. Consequently, standardized grading protocols, when merged with advanced sorting technologies, play an indispensable role in maintaining postharvest quality, increasing shelf life, and maintaining the commercial value of jujube fruits from farm to consumer.

### 3.3. Cleaning and Sanitization

Cleaning and sanitization are censorious postharvest practices for jujube, with a focus on removing dirt, pesticide residues, and pathogenic microorganisms that lead to spoilage. Standard practice generally involves washing fruits with potable water, often supplemented with sanitizing treatments to prolong shelf life [48]. Chlorine solutions at 100 ppm have demonstrated notable effectiveness, reducing fungal infections by up to 80% and significantly delaying fruit decay. Nonetheless, concerns towards chemical residues and potential health risks have stimulated interest in alternative, safer methods.

Ozone treatment has come out as a promising chemical-free alternative, proficient at effectively reducing microbial loads while simultaneously boosting antioxidant capacity. Likewise, UV-C irradiation has been applied for surface disinfection. Although UV-C treatment successfully limits microbial contamination, it may also influence quality attributes such as firmness and color retention [49]. Thus, to maintain the safety and marketability of jujube fruits, sanitization techniques are essential and choice of treatment must maintain microbial control with the preservation of sensory and nutritional quality.

### 3.4. Packaging Systems

Packaging plays a vital role in maintaining the quality of jujube fruits throughout storage, transportation, and marketing. Its primary function is to retain moisture, preventing dehydration and preserving fruit freshness and quality. By reducing transpiration and moisture loss, packaging helps minimize weight reduction and maintain firmness [49]. Adequate ventilation is necessary to prevent excessive humidity buildup, which can otherwise promote mold and bacterial growth. Thus, packaging systems should be carefully designed to balance moisture retention with proper gas exchange and help optimal storage conditions.

To extend shelf life and maintain fruit quality, advanced packaging technologies such as MAP and CA systems have proven effective [50]. MAP, specifically, has been widely applied to jujube fruits due to their high perishability. This method alters the surrounding atmosphere by lowering oxygen levels and increasing carbon dioxide concentrations, and by slowing respiration and delaying ripening. Micro-perforated MAP is especially suitable for jujubes as it allows effective regulation of gas exchange and prevents anaerobic conditions [22]. Auxiliary MAP approaches include vacuum packaging and gas flushing with inert gases such as nitrogen [22].

MAP systems can also be mixed with complementary preservation techniques for enhanced effectiveness. For instance, aloe vera gel coatings or calcium chloride dips combined with MAP have been reported to further delay senescence and maintain quality [21]. Calcium treatment strengthens cell walls by cross-linking pectin molecules, stabilizes membrane integrity, and reduces the activity of cell wall-degrading enzymes, thereby slowing tissue softening and delaying fruit aging [51]. Additionally, the category of container used, whether plastic or cardboard boxes, can influence the effectiveness of packaging in reducing moisture loss and physical damage [52]. In general, MAP, particularly with micro-perforations, stands out as a key technology for extending the shelf life and maintaining the commercial quality of jujube fruits, especially when used together with other postharvest treatments.

### 3.5. Postharvest Monitoring and Quality Assurance

Effective postharvest monitoring is essential for maintaining the quality, safety, and marketability of jujube fruits during storage and transportation. This involves a combination of real-time environmental sensing and periodic fruit quality assessments based on chemical, microbiological, and physical metrics. Real-time sensors are increasingly applied in this process to thoroughly pursue key environmental parameters such as temperature, humidity, and gas composition. Temperature monitoring ensures that fruits are maintained within the optimal range, thereby slowing ripening and inhibiting microbial growth. Humidity sensors assist in sustaining the ideal moisture balance, limiting both condensation and excessive dehydration. Gas composition sensors monitor oxygen and carbon dioxide concentrations, which can be maintained through MAP to increase shelf life and delay senescence. Consequently, sensing technologies generate valuable real-time data that enable immediate adjustments to storage conditions, certifying that fruits remain fresh and of high quality.

Beyond environmental control, routine fruit sampling is necessary to evaluate quality decline. Standard assessments include physical, chemical, and microbiological quality. Physical fruit firmness is quantitatively measured using a penetrometer (e.g., Magness-Taylor probe) and expressed in Newtons (N) or kgf. Weight loss, a critical indicator of postharvest deterioration, is calculated as a percentage of initial weight. Visual quality is often scored based on standardized scales for skin color (using colorimeters like a Chroma Meter for *L*, *a*, and *b* values), shine, and the incidence of shriveling, browning, or surface pitting. The TSS content, indicative of sugar levels, is measured with a digital refractometer and expressed as °Brix. TA, representing organic acid content, is determined via titration with NaOH and results are expressed as a percentage of malic or citric acid. The TSS/TA ratio is a key determinant of flavor balance. The concentration of ascorbic acid (vitamin C), a vital nutrient highly susceptible to degradation, is commonly assessed using spectrophotometric methods (e.g., 2,6-dichlorophenolindophenol titration). Antioxidant capacity and total phenolic content are frequently evaluated using assays such as DPPH/FRAP and the Folin–Ciocalteu method, respectively, to track the retention of bioactive compounds. Safety and spoilage are monitored by enumerating microbial loads. Standard plate counts on agar media (e.g., Plate Count Agar for aerobic mesophilic bacteria, Potato Dextrose Agar for yeasts and molds) are used to assess total viable counts and identify specific spoilage organisms, ensuring levels remain within safe and acceptable thresholds.

## 4. Storage Technologies for Shelf-Life Extension

**Figure 2:**
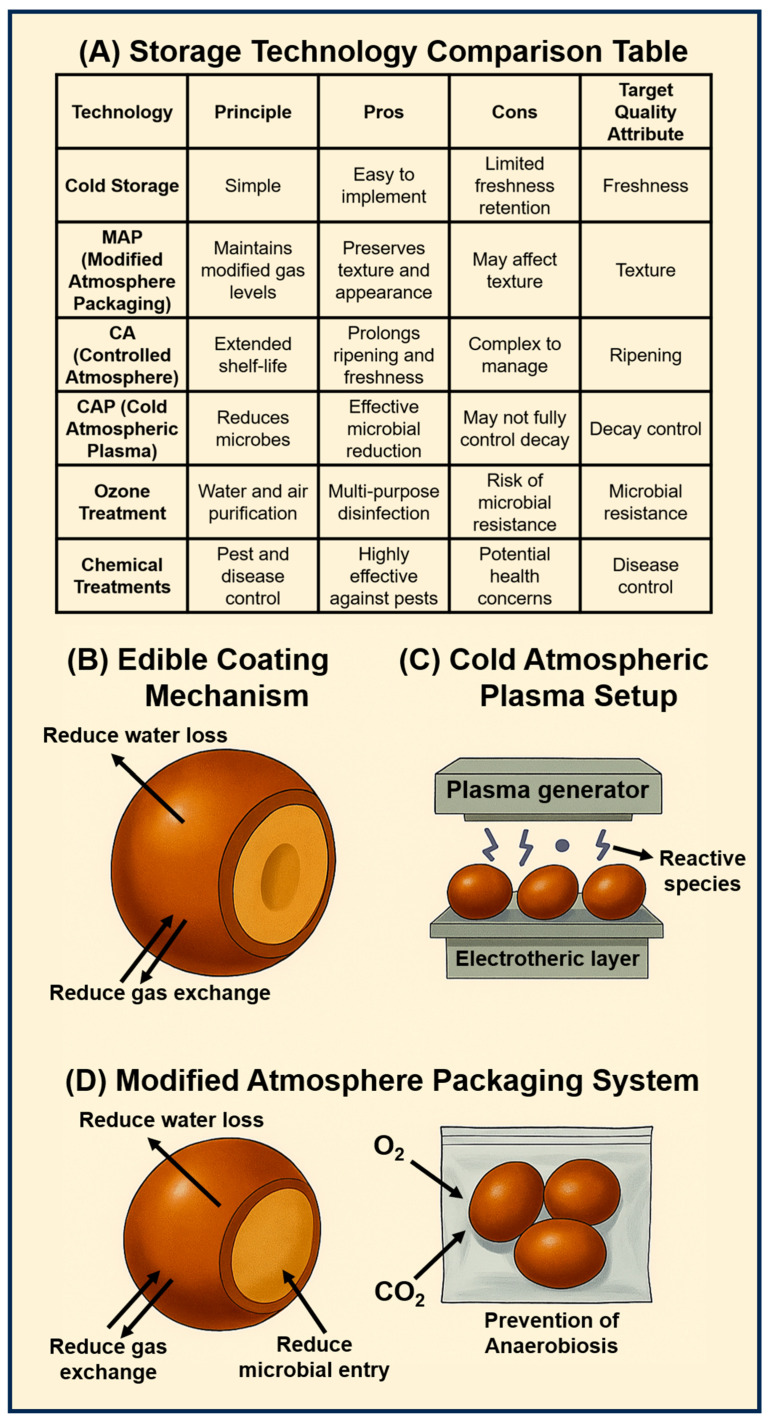
Storage technologies for postharvest preservation of jujube fruit.

Effective storage technologies are essential to slow down the rapid physiological deterioration of jujube and extend its marketability. Conventional methods such as cold storage, MAP, and CA storage play a central role in reducing respiration, decay, and nutrient loss. In parallel, natural approaches like edible coatings and aloe vera gel provide protective barriers against moisture loss and microbial spoilage, while emerging innovations such as smart packaging and non-thermal technologies (e.g., UV-C, pulsed light, and ozone) offer sustainable and consumer-friendly alternatives. A concise overview of these conventional and emerging postharvest strategies is summarized in Table 1. The principles and quality attributes of different storage technologies with their pros and cons are mentioned in Figure 2A.

### 4.1. Cold Storage

One of the most commonly used postharvest technologies in extending the shelf life of the jujube fruits is cold storage, which is effective in slowing down physiological and biochemical activity like respiration, ethylene biosynthesis, and growth of microbes. Traditional cold storage facilities often have temperatures of 0 °C to 10 °C with relative humidity widely regulated at 85–95%. Standard implementations mainly include cold rooms and mechanical refrigeration, both of which inhibit microbial spoilage and slow down the enzyme-driven senescence [53].

Recent research has highlighted the ideal conditions of storage of jujube as 0–1 °C and 90 ± 5% RH. These parameters result in considerably less weight loss, firmness, and chilling injury than higher temperatures of 10–15 °C or ambient conditions [53,54]. Low temperatures inhibit metabolic activities along with enzymatic activity, respiration, and ethylene production. The work of cell wall-softening enzymes, like polygalacturonase, are specifically suppressed and fruit texture and structural integrity are maintained. Moreover, storage at low temperatures decreases the presence of fungal pathogens like *Rhizopus* and *Colletotrichum*, which are the key causes of postharvest losses in jujube [55]. Storage at 10 °C enabled Indian jujube to retain quality and had a storage span of 30–35 days without much loss of weight and minimal chilling injury when compared to the rapid degradation of fruits stored at ambient temperature [54]. Positive results have been also observed in Xinjiang, China, and South Punjab, Pakistan, where the use of high-tech refrigerated storage silos has significantly prolonged shelf life and enhanced the quality of the fruits in general [56]. These results validate the use of cold storage as one of the developable strategies in the current jujube supply chain to offer stable quality and marketability.

### 4.2. Modified Atmosphere Packaging (MAP)

An efficient postharvest technology that can greatly increase the shelf life and preserve the quality of jujube fruits is modified atmosphere packaging (MAP). Figure 2 illustrates conventional and emerging storage approaches used to extend fruit shelf life and maintain quality. MAP extends ripening through the control of the gas content in the package, controls weight loss, and preserves firmness, color, and nutrition. MAP can be used passively whereby respiration of fruits alters the internal atmosphere, or it may be used actively via flushing the package with gas mixtures (Figure 2D).

Micro-perforated films (PMP-MAP) and laser-microporous MAP offer a precise gas exchange, and thus are highly applicable to the high respiration rate of jujube [57,58]. Most packaging materials are low-density polyethylene (LDPE), polyethylene (PE), and multilayer or perforated films, which vary in terms of gas permeability and ability to retain moisture [53,57]. The ideal MAP environment usually limits oxygen to approximately 3–10% and increases carbon dioxide to less than 5%, and thereby slows down ripening, minimizes water loss, and inhibits the growth of microbes. This is a critical gas balance, especially to the high-breathing fruits like jujube. PMP-MAP and laser-microporous films are highly beneficial because they can enable excess CO_2_ to escape at the same time as adequate O_2_ is available, preventing the process of anaerobic fermentation and off-flavor formation [57,58].

MAP has been reported to significantly decrease decay and weight loss and preserve antioxidants and ascorbic acid in jujube cvs. like ‘Li’ and ‘Dongzao’ [53]. For instance, jujube fruits of cv. ‘Phoenix’ stored in MAP at 5 °C and 90% relative humidity retained their appearance even after 49 days [49]. The shelf life of MAP treatments using commercial wraps (e.g., Fresh MAP) was found to be 60 days at 0 ± 0.5 °C and 90 ± 5% RH, and the best wraps reduced weight and vitamin C losses [59]. Furthermore, PMP-MAP is reported to lessen the winter jujube respiration rates to preserve the total soluble solids (TSS) and titratable acidity, and to postpone the beginnings of reddening and decay [57]. Taken together, these results show that MAP is a powerful and diverse technology for prolonging storage duration and preserving the postharvest quality of jujube fruits.

### 4.3. Controlled Atmosphere (CA) Technology

CA storage refers to the constant maintenance of the gaseous environment around the fruits to slow down respiration, ripening, and microbial spoilage. Unlike MAP, which is primarily package-based, CA systems are large-scale facilities equipped with gas analyzers and automated feedback systems that maintain specific O_2_ and CO_2_ concentrations (and humidity in advanced systems) within sealed storage chambers [60].

The optimal CA conditions for jujube typically include O_2_ levels between 2 and 5% and CO_2_ concentrations below 2%, effectively inhibiting enzymatic activity and microbial growth. For instance, storage under 3% O_2_ + 0% CO_2_ preserved ascorbic acid, reduced MDA (a marker of lipid peroxidation), and maintained cell membrane integrity. Another study reported that jujubes stored at 5 kPa O_2_ + 10 kPa CO_2_ for 37 days at 5 °C, followed by five days at 15 °C, retained higher soluble solids content and titratable acidity compared with controls. Similarly, CA storage conditions of 3–5% O_2_ and 5–8% CO_2_ preserved firmness, TSS, and total phenolic content during the first eight weeks of storage while reducing oxidative damage and slowing bioactive compound degradation [49].

According to [5], incorporating CA storage into postharvest handling protocols is more effective than conventional cold storage alone, as it modulates cellular metabolic activity and further delays senescence. Thus, CA technology represents a highly effective approach for maintaining nutritional integrity, reducing oxidative damage, and extending the postharvest life of jujube fruits.

### 4.4. Cold Atmospheric Plasma and Ozone Treatment

CAP technologies, including DBD, APPJ, and CDPJ, are emerging non-thermal approaches that generate reactive oxygen and nitrogen species (O, OH, NO), along with UV photons, at near-ambient temperatures. These reactive agents not only exhibit strong antimicrobial activity but also modulate physiological and metabolic processes in fruits [61,62]. Plasma can be applied directly to fruit surfaces or in-package (e.g., volumetric DBD), with or without the addition of carrier gases such as air, O_2_, N_2_, Ar, or He (Figure 2C) [61].

Ozone (O_3_), applied either as a dry gas or dissolved in water (aqueous ozone), is another powerful oxidizing agent widely used to sanitize fruits by inactivating pathogens and reducing ethylene levels. Application methods include batch treatments in sealed chambers or intermittent low-dose exposure in storage facilities [41,56,63]. Both plasma and ozone treatments extend shelf life by inhibiting surface microbes, degrading ethylene to delay ripening, and stimulating cellular responses that enhance antioxidant potential.

Plasma treatment has been reported to reduce or delay softening by inactivating cell wall-modifying enzymes such as polygalacturonase, thereby limiting pectin solubilization and maintaining tissue firmness [62,64]. In the same manner, 20 min of exposure to 25 ppm of ozone lowered weight loss, inhibited decay, and elevated phenolic content and antioxidant activity in jujube analogues [63]. Recently, it was shown that ‘Dongzao’ cv. of jujube fruit could be successfully treated by cold plasma (10–20 min) to inhibit decay and maintain quality properties throughout cold storage. Notably, ozone and plasma treatments do not leave residues, are friendly to the environment, and can be used with the current cold-chain logistics, which increases their commercial relevance [62,65].

### 4.5. Novel/Non-Thermal Technologies

Besides cold plasma and ozone, other new non-thermal technologies, mainly X-ray irradiation, high-intensity ultrasound, gamma irradiation, EBI, and pulsed electric field PEF, are being investigated to extend the postharvest life of jujube fruits by preserving their quality. CAP has demonstrated the most encouraging effects among them, where 20 min treatment of winter jujube kept at 4 °C with 90% RH greatly delayed the onset of ripening, reduced the population of microorganisms, increased the total phenolic compounds and antioxidant activity, and decreased the level of oxidative stress indicators, including H_2_O_2_ and malondialdehyde [42,57,66]. Low-dose X-ray irradiation (≈0.3 kGy) has also proved effective, and prolonged the shelf life of winter jujube stored at ambient conditions by up to 12 days, decreased fungal growth by an average of 8%, and decreased aerobic microbial loads by an average of 29% [67].

Other irradiation methods, including gamma rays and EBI, have been successfully applied in pest and pathogen control and shelf-life extension across a wide range of fresh produce, though targeted studies on jujube remain limited [66,68,69]. PEF technology represents another promising approach. This method applies short, high-voltage pulses that disrupt microbial cell membranes, achieving microbial inactivation while preserving aroma, flavor, and nutritional quality, thereby improving physico-chemical attributes [70]. High-intensity ultrasound has similarly demonstrated effectiveness in improving microbial safety and stimulating antioxidant responses in fruits such as litchi, strawberry, and kiwifruit [71,72,73]. Although direct applications to jujube are currently scarce, the positive results in related fruits suggest considerable potential for future adaptation. Collectively, these non-thermal technologies provide versatile, residue-free, and energy-efficient preservation methods. By delaying senescence and decay, while maintaining nutritional and sensory qualities, they hold significant promise for enhancing the postharvest shelf life and market value of jujube fruits.

Despite their promising potential, non-thermal technologies for fruit preservation also have several limitations. Treatments such as cold plasma, ozone, and irradiation require specialized equipment and technical expertise, which can increase operational costs and limit scalability for commercial use. Some methods, including high-intensity ultrasound, gamma irradiation, and electron beam irradiation, may cause uneven treatment or partial quality degradation if not carefully optimized. Additionally, targeted studies on jujube remain limited, and long-term effects on sensory attributes, nutritional compounds, and consumer acceptance are not fully understood. Regulatory restrictions and public perception regarding irradiation-based methods may further constrain their widespread adoption.

### 4.6. Edible Coatings

Edible coatings are thin layers of edible materials applied to fruit surfaces that act as semi-permeable barriers, reducing water loss, regulating gas exchange, and, in some cases, serving as carriers for bioactive or antimicrobial agents (Figure 2B). They are typically classified according to the primary biopolymer used: polysaccharides (e.g., chitosan, pectin, cellulose), proteins (e.g., gelatin, soy, whey), and lipids (e.g., waxes, oils). More recently, herbal and composite coatings such as chitosan combined with aloe vera have gained attention for jujube preservation [74]. The primary mode of action of edible coatings is to reduce transpiration by forming a semi-permeable barrier, thereby minimizing water loss, weight reduction, and shriveling. By restricting oxygen entry and slowing ethylene diffusion, coatings also modulate fruit metabolism, delaying ripening and senescence [75]. Coatings based on chitosan (1%) combined with cinnamon oil (0.1%) have demonstrated exceptional performance in reducing weight loss and decay during cold storage. Fruits treated with this formulation exhibited only 0.53% weight loss and 13.83% decay after 60 days at 4 °C, representing reductions of 62% and 67%, respectively, compared to uncoated controls [76]. This coating also preserved critical quality attributes, including vitamin C content (3.08 mg/g in coated fruits versus 2.55 mg/g in controls) and titratable acidity (0.342%), highlighting its ability to maintain both nutritional and organoleptic properties over extended storage. Similarly, aloe vera gel coatings, applied at concentrations of 33% and 50% *v*/*v*, significantly reduced weight loss (by approximately 30%) and decay rates during 40 days of refrigerated storage, while pectin coatings at 1.5% *w*/*v* achieved comparable results [28]. When combined with 5% ascorbic acid, aloe vera coatings also effectively preserved fruit quality under ambient conditions, reducing weight loss by 46% (14.69% vs. 27.35% in controls) and maintaining higher levels of titratable acidity and antioxidant activity over 15 days [77].

Composite coatings have further enhanced fruit preservation. Sodium alginate (2%) with olive oil (0.2%) enriched with antioxidants effectively limited weight loss, stabilized total soluble solids (TSS), and maintained antioxidant content during ambient storage at 65% relative humidity [78]. Xanthan gum (0.3%) coatings reduced weight loss relative ion leakage and malondialdehyde (MDA), superoxide anion, and hydrogen peroxide (H_2_O_2_) contents, and delayed TSS accumulation, thus slowing ripening and extending storage life by up to 15 days. Additionally, coatings with carboxymethyl cellulose and pullulan not only reduced physiological losses but also maintained higher levels of bioactive compounds and enzymatic activity, further contributing to overall fruit quality during storage. The beneficial effects of edible coatings extend beyond physical and chemical preservation. They positively modulate enzymatic defense mechanisms, enhancing the activity of SOD, POD, and PPO, thereby improving antioxidant capacity and mitigating oxidative stress. For instance, chitosan–cinnamon oil coatings increased SOD activity to 14.53 U/g compared to 9.07 U/g in control fruits and maintained POD and PPO activities at 63.6 U/g and 13.40 U/g, respectively [76]. Coatings enriched with functional additives, such as tea polyphenols and ascorbic acid, have been shown to further sustain enzymatic activities and total phenolic content under ambient storage conditions [27,77].

In addition to slowing physiological and biochemical degradation, edible coatings serve as effective barriers against microbial contamination, delaying spoilage and enhancing the visual and nutritional quality of jujube fruits. Coatings can be fortified with antimicrobial agents such as essential oils or natamycin, which suppress spoilage organisms and enhance safety [79]. Their multifaceted effects, combining moisture regulation, microbial inhibition, and enhancement of biochemical defenses, make edible coatings a highly promising postharvest technology for ensuring the prolonged quality and marketability of jujube fruits under diverse storage conditions. Lipid-based coatings such as carnauba wax further improve surface gloss, enhancing visual appeal and consumer acceptance [80].

### 4.7. Chemical Treatments

Chemical dipping and vapor treatments have long been used in postharvest preservation of fruits, primarily to delay senescence, strengthen cell wall integrity, and inhibit fungal and bacterial infections. Commonly applied agents include calcium salts (chloride, nitrate, sulfate), plant hormones (such as 1-MCP), ascorbic acid, preservatives, natamycin, melatonin, and mineral mixes [81]. For jujube, chemical treatments focus on enhancing firmness, delaying ripening, and reducing decay. By providing firmness and decay suppression, calcium salts (0.5–4%), such as calcium chloride, calcium nitrate and calcium sulfate, stiffen the cell wall structure [82,83]. The ethylene inhibitor 1-MCP is highly effective in delaying the ripening and senescence without affecting firmness, vitamin C content, or antioxidant properties [81]. Surface sanitizers such as natamycin (antifungal) and propyl gallate (antioxidant) prolong shelf life by minimizing microbial contamination and oxidative stress [41]. Immune-priming compounds such as oligochitosan and BABA strengthen the fruit’s defense responses, thereby reducing postharvest rot [42]. Among further processing techniques, acidic electrolyzed water and organic acids (such as ascorbic and citric acid) exhibit broad-spectrum antimicrobial activity and act as safe, non-toxic sanitizers that also help reduce browning [84].

**Figure 3:**
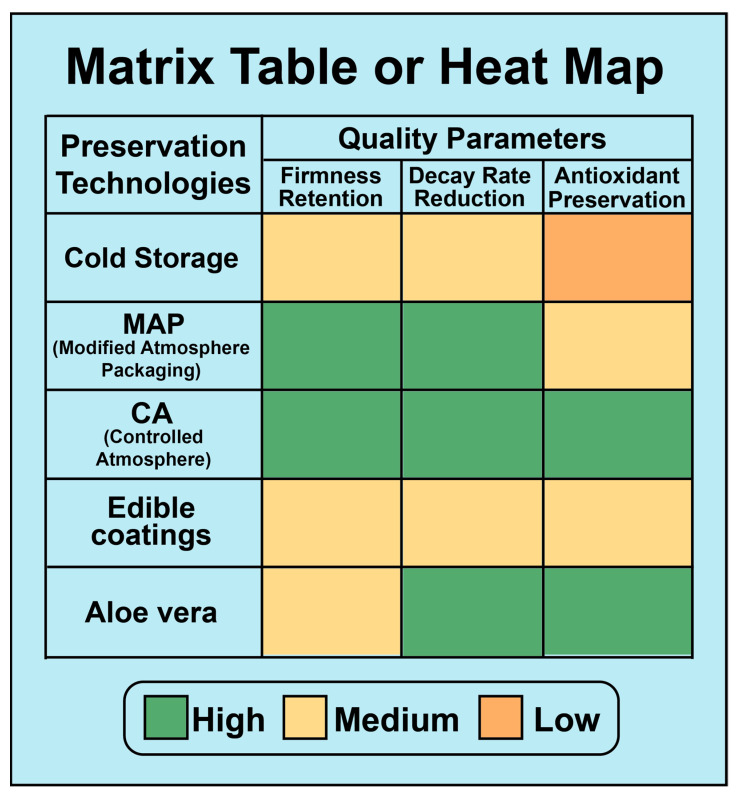
Functional overview of postharvest preservation strategies.

The processes involved in these chemical treatments are due to several physiological and biochemical pathways. Firmness retention occurs when the calcium reacts with the pectin and produces cross-links in the cell wall, slowing the softening process and increasing resistance to spoilage. Antimicrobial defenses include compounds such as natamycin, which disrupt fungal cell membranes, and elicitors like oligochitosan and BABA, which activate systemic resistance mechanisms in the fruit [42]. The mechanism of ethylene suppression involves irreversible binding of 1-MCP to ethylene receptors to inhibit downstream ripening signals regardless of pre- or postharvest application [59]. Thus, chemical treatments remain a cornerstone of jujube postharvest management, but the related procedures need to be optimized to have an impact on both safety and consumer acceptance. Figure 3 presents a matrix table regarding the impact of different postharvest preservation techniques on fruit quality.

**Table 1:** Overview of conventional and emerging preservation postharvest strategies.

Category	Types/Material	Examples (Including Coating Agents, Chemicals, Non-Thermal Devices, etc.)	Main Effects/Purpose	Key Citations
Cold storage	Refrigerated storage (0–4 °C)	Cold storage at 0–4 °C for winter/Chinese jujube	Slows respiration, reduces weight loss and decay; delays reddening/senescence	[29]
MAP	Passive/active MAP (O_2_/CO_2_/N_2_)	MAP at ~15–25% O_2_ and 5–10% CO_2_; vacuum vs. active MAP	Lowers respiration and browning; extends shelf life and maintains firmness/flavor	[22]
	Micro-perforated films (PMP-MAP)	Laser micro-perforated BOPP for winter jujube	Prevents anaerobiosis, preserves antioxidants and flavor; reduces reddening/decay	[22]
	AEW + MAP combo	AEW rinse + MAP	Maintains firmness, delays softening; enhances antioxidant system	[30]
CA	Low O_2_/low CO_2_	3–5% O_2_, <2% CO_2_, 2–0 °C, RH >95%	Extends storage (2–4 months reported); reduces decay and maintains quality	[51]
	Elevated oxygen (≈60% O_2_)	60% O_2_ atmosphere for winter jujube at 0–4 °C	Maintains antioxidant capacity; inhibits anaerobic metabolism and off-flavor	[30]
Novel/non-thermal technology	CAP	DBD/plasma jet exposure	Reduces weight loss/decay; delays reddening and senescence; improves ROS balance	[26]
	Ozone (gaseous)	2.5–10 μL/L O_3_ exposure	Reduces decay by modulating fruit-surface microbiome	[56]
	Ozone (aqueous)	1.5–3 mg/L dissolved O_3_ wash	Decontamination with minimal quality impact; extends shelf life	[53]
	UV-C (photochemical)	5 kJ/m^2^ UV-C; storage at 4 °C	Delays senescence; regulates ROS and phenylpropanoid metabolism; lowers decay	[62]
	Ultrasound-assisted sanitizing	Ultrasound + low-chlorine wash	Prevents cross-contamination; effective decontamination without hurting quality	[32]
	UV-C + biocontrol	UV-C + *Metschnikowia pulcherrima* yeast	Controls *Alternaria* rot; reduces decay without quality loss	[67]
Edible coatings	Chitosan (CTS)	1% chitosan coating	Reduces weight loss, decay; preserves firmness	[20]
	Chitosan + cinnamon oil	1% CTS + 0.10–0.75% cinnamon oil	Antifungal, lowers decay and weight loss; maintains sensory quality	[76]
	Aloe vera gel	33–50% Aloe vera	Cuts weight loss; preserves TSS/TA; better firmness	[28]
	Pectin	1.0–1.5% pectin	Reduces weight loss; maintains acidity	[28]
	CMC	1–2% carboxymethyl cellulose	Delays browning and firmness loss	[28]
	Composite CTS/nano-SiO_2_/alginate	CTS + nano-SiO_2_ + alginate blend	Improves moisture barrier; prolongs shelf life	[13]
	Pectin + natamycin	Pectin matrix + natamycin	Controls decay while maintaining quality	[53]
	Chitosan + ε-PL	1% CTS + ε-PL	Enhanced decay suppression; maintains firmness	[85]
Chemical treatments	1-MCP	0.5–1.0 μL/L 1-MCP	Delays ripening/senescence; reduces decay	[85]
	1-MCP + CaCl_2_	1-MCP + 1–2% CaCl_2_	Synergistic firmness and quality retention	[85]
	1-MCP + SA	1-MCP + salicylic acid	Best shelf-life extension via ROS regulation	[86]
	Salicylic acid	1–2 mM SA	Controls Alternaria rot; induces resistance	[20]
	Calcium chloride	1–2% CaCl_2_	Supports cell wall integrity	[20]
	Calcium nitrate	1% Ca(NO_3_)_2_	Maintains firmness and quality	[83]
	SNP	SNP fumigation	Delays senescence; lowers browning	[16]
	NaHS	NaHS fumigation	Extends storage; regulates ROS	[7]
	Gibberellin (GA_3_)	≈10 mg/L GA_3_	Delays reddening; slows senescence	[87]
	Brassinolide (BR) ± CaCl_2_	BR + CaCl_2_	Maintains quality; reduces decay	[16]
	Ascorbic acid	1–2% AA dip	Antioxidant; maintains firmness	[17]
	Citric acid	0.5–1.5% CA	Reduces decay; maintains sugars/TA	[16]

## 5. Challenges and Research Gaps

Postharvest handling of jujube has advanced considerably, yet significant challenges remain to ensure minimal losses across the supply chain. The thin epidermis of jujube makes it highly perishable, with a high respiration rate and vulnerability to mechanical damage, which accelerates degradation. Common postharvest issues such as enzymatic browning, water loss, and microbial decay are particularly severe under improper temperature and humidity control. Variations among cultivars in terms of physiology and ripening further complicate standardization of handling and storage practices. Mechanical harvesting, while reducing dependence on manual labor, increases the risk of physical damage and accelerated spoilage. The widespread use of chemical preservatives raises concerns regarding consumer safety, particularly the potential accumulation of harmful residues on the fruit. Regulatory restrictions and growing consumer demand for “clean-label” foods emphasize the need for alternatives that ensure safety without compromising quality. Sustainable postharvest strategies, such as natural antimicrobial coatings, edible films, or non-thermal processing methods, offer a pathway to minimize chemical residues while maintaining microbial control. Infrastructure limitations, especially in developing regions, hinder adoption of advanced technologies like controlled-atmosphere storage or sensor-based monitoring. Additional losses are linked to insufficient farmer training, inadequate packaging, and disruptions in transportation and marketing.

Several critical research gaps remain in improving postharvest preservation. There is limited understanding of cultivar-specific postharvest physiology, including differences in respiration rates, ripening patterns, and susceptibility to microbial decay, which restricts the development of targeted preservation strategies. Non-thermal technologies such as cold atmospheric plasma, irradiation, pulsed electric fields, and high-intensity ultrasound are promising, but their effects on long-term nutritional quality, sensory properties, and consumer acceptance require further study. Synergistic approaches, such as combining edible coatings with modified atmosphere packaging, remain underexplored with respect to scalability, economic feasibility, and environmental sustainability. Integration of smart packaging and IoT-enabled real-time monitoring into commercial supply chains also needs validation under diverse climatic and logistical conditions. Finally, identification of natural, biodegradable antimicrobial and antioxidant compounds that are as effective as synthetic chemicals is crucial for reducing chemical residues, ensuring food safety, and supporting sustainable production. Socio-economic feasibility studies evaluating adoption, trade-offs, and supportive policies are limited, restricting large-scale implementation of sustainable postharvest technologies.

## 6. Conclusions

Jujube is highly valued for its nutritional, medicinal, and economic significance, yet it remains highly perishable due to its thin epidermis, high respiration rate, and susceptibility to damage. This review offers a novel integrative perspective, combining conventional storage methods, such as cold storage, MAP, and CA, with emerging technologies including plasma, ozone, edible coatings, non-thermal treatments, and smart packaging, providing a comprehensive overview of strategies that extend shelf life and preserve fruit quality. Combined approaches, such as MAP with coatings, have shown improved outcomes, where interactions may be additive or, in some cases, synergistic. Despite these advances, challenges persist, including cultivar-specific physiology, limited scalability of certain non-thermal methods, consumer concerns over preservatives, and gaps in cold-chain infrastructure. Given the growing market demand and economic value of jujube, optimizing postharvest strategies is essential to ensure quality, extend market reach, and enhance profitability. Future research should focus on cultivar-specific protocols, natural preservatives, IoT-based monitoring, and socio-economic analyses to develop sustainable, commercially viable solutions that strengthen jujube preservation and global supply chains.

## Data Availability

No new data were created or analyzed in this study. Data sharing is not applicable to this article.

## List of Abbreviations

The following abbreviations are used in this manuscript:

1-MCP	1-Methylcyclopropene
AA	Ascorbic Acid
AEW	Acidic Electrolyzed Water
APPJ	Atmospheric Pressure Plasma Jet
BABA	β-Aminobutyric Acid
BOPP	Biaxially Oriented Polypropylene
BR	Brassinolide
CA	Controlled Atmosphere
Ca(NO_3_)_2_	Calcium Nitrate
CaCl_2_	Calcium Chloride
CAP	Cold Atmospheric Plasma
CDPJ	Corona Discharge Plasma Jet
CMC	Carboxymethyl Cellulose
CTS	Chitosan
Cx	Cellulase
DBD	Dielectric Barrier Discharge
DPPH	2,2-Diphenyl-1-picrylhydrazyl (radical scavenging assay)
EBI	Electron Beam Irradiation
FRAP	Ferric Reducing Antioxidant Power
GA_3_	Gibberellic Acid
LDPE	Low-Density Polyethylene
MAP	Modified Atmosphere Packaging
MDA	Malondialdehyde
NaHS	Sodium Hydrosulfide (Hydrogen Sulfide donor)
O_3_	Ozone
PE	Polyethylene
PEF	Pulsed Electric Field
PG	polygalacturonase
PMP-MAP	Perforated Micro-Perforated Modified Atmosphere Packaging
POD	Peroxidase
PPO	Polyphenol Oxidase
ROS	Reactive Oxygen Species
SA	Salicylic Acid
SNP	Sodium Nitroprusside (Nitric Oxide donor)
SOD	Superoxide Dismutase
TA	Titratable Acidity
TSS	Total Soluble Solids
UV-C	Ultraviolet-C
α-L-Af	α-L-Arabinofuranosidase
β-Gal	β-Galactosidase
β-Glu	β-Glucosidase
ε-PL	ε-Polylysine
